# The gut microbiota in cancer immunity and immunotherapy

**DOI:** 10.1038/s41423-025-01326-2

**Published:** 2025-08-06

**Authors:** Mingxu Xie, Xiang Li, Harry Cheuk-Hay Lau, Jun Yu

**Affiliations:** 1 Institute of Digestive Disease, Department of Medicine and Therapeutics, State Key Laboratory of Digestive Disease, Li Ka Shing Institute of Health Sciences, CUHK Shenzhen Research Institute, The Chinese University of Hong Kong, Hong Kong SAR, China; 2 Center for Gut Microbiome Research, Med-X Institute Centre, The First Affiliated Hospital of Xi’an Jiaotong University, Xi’an, Shaanxi China; 3 Department of Otorhinolaryngology-Head and Neck Surgery and Center for Gut Microbiome Research, The First Affiliated Hospital of Xi’an Jiaotong University, Xi’an, Shaanxi China

**Keywords:** Gut microbiota, Cancer immunity, Immunotherapy response, Microbial modulation strategies, Tumor microenvironment, Cancer microenvironment, Cancer immunotherapy

## Abstract

The human gastrointestinal tract harbors trillions of microorganisms, including bacteria, fungi, and viruses, to form the gut microbiota. Cumulative evidence has demonstrated the critical impact of gut microbes on cancer immunity. In cancer, an altered gut microbiota enriched with pathogenic bacteria can actively promote immune evasion and disrupt antitumor immunity, thereby supporting tumor growth and survival. Conversely, beneficial commensal bacteria (e.g., *Lactobacillus* and *Bifidobacterium*) have emerged as therapeutic probiotics for cancer prevention and as adjuvants for cancer therapy. The gut microbiota is also closely linked to the efficacy of immunotherapy. This review summarizes the effects of pathogenic bacteria and beneficial commensals, including T cells, B cells, natural killer cells, innate lymphoid cells, and myeloid-derived suppress cells, on various innate and adaptive immune cell populations in cancer. It also explores the mechanisms by which the gut microbiota influences immunotherapy efficacy, such as the modulation of innate immune cells and CD8^+^ T cells. Given its importance, an increasing number of studies have developed approaches to target the gut microbiota to improve immunotherapy outcomes and reduce immune-related adverse events. These strategies include antimicrobial intervention, probiotics, prebiotics/dietary modifications, microbial metabolites, phage therapy, and fecal microbiota transplantation. This review also evaluates clinical applications that use the gut microbiota to predict immunotherapy outcomes. Overall, the current understanding of host‒microbe interactions within the tumor microenvironment has laid a critical foundation for the translation of microbiota research into clinical practice, ultimately benefiting patients.

## Introduction

Cancer development is closely linked to immune surveillance evasion, where tumor cells exploit intricate mechanisms against host antitumor defenses, including defective antigen presentation, immune checkpoint activation, and the recruitment of immunosuppressive cells. The recent emergence of immunotherapy, particularly immune checkpoint blockade (ICB), has transformed cancer treatment. ICB, which targets programmed cell death protein (PD-1; e.g., pembrolizumab and nivolumab), its ligand programmed cell death ligand 1 (PD-L1; e.g., atezolizumab and durvalumab), or their combination with cytotoxic T-lymphocyte associated protein 4 (CTLA-4) antagonists (e.g., ipilimumab and tremelimumab), has demonstrated robust efficacy in treating metastatic melanoma (MM), renal cell carcinoma (RCC), and non-small cell lung cancer (NSCLC) through reinforcing and reactivating antitumor immunity [1]. However, many cancer types are characterized as “cold tumors”, which have limited intratumoral infiltration of tumor-killing immune cells, thus markedly reducing ICB efficacy [2]. As a result, clinical studies have reported that ICB is effective in only approximately half of cancer patients [3, 4]. Such treatment heterogeneity arises not only from tumor genomics but also from the host microenvironment, including intratumoral infiltration of immune cells and the gut microbiota [5].

The gut microbiota is now emerging as a critical determinant in cancer immunotherapy. An increasing number of studies have reported that these gut microbes not only influence immunotherapy efficacy but also yield the capacity to improve treatment outcomes [6–9]. For example, phase I-II clinical trials of fecal microbiota transplantation (FMT) from ICB responders to nonresponders have shown that this approach can restore sensitivity to anti-PD-1 treatment in patients with MM [10, 11]. Similarly, another phase I trial reported that transferring stools from healthy donors achieves an improved response rate to PD-1 inhibitors in recipient patients with advanced MM [12]. Targeting the gut microbiota by FMT also alleviates adverse events associated with immunotherapy [13] (Table 1). Given its importance, current research has focused on developing gut microbiota-targeting therapeutic approaches [14, 15]. Nonetheless, the precise mechanisms of microbial interventions in cancer immunotherapy remain unclear. In this review, we explore the interplay and underlying mechanisms between the gut microbiota and immune cells in the TME and their translational implications for cancer immunotherapy. The clinical potential of microbiota-targeting strategies to improve immunotherapy efficacy (e.g., FMT, probiotics, prebiotics/dietary interventions) and the application of microbial biomarkers to predict treatment outcomes are also discussed.

**Table 1:** Clinical trials of fecal microbiota transplantation in cancer immunotherapy

Cancer type	Study number	Phase	Immunotherapy	Enrollment	Country
Head and neck squamous cell carcinoma, Cutaneous squamous cell carcinoma, Clear cell renal cell carcinoma, Melanoma, Non-small cell lung cancer	NCT05286294	Phase 2	ICB	20	Norway
stage IV cancer	NCT05273255	NA	ICB	18	Switzerland
Solid tumor	NCT04264975	NA	ICB	60	South Korea
Liver cancer	NCT05690048	Phase 2	Atezolizumab	48	Germany
Melanoma	NCT03353402	Phase 1	PD-1 blockade	40	Israel
Lung cancer	NCT04924374	NA	Pembrolizumab, Nivolizumab, Atezolizumab	25	Spain
Renal cell carcinoma	NCT04163289	Phase 1	Ipilimumab, Nivolumab	20	Canada
Metastatic lung cancer	NCT05502913	Phase 2	ICB	80	Israel
Melanoma	NCT05251389	Phase 1, 2	ICB	24	Netherlands
Gastrointestinal cancer	NCT04130763	Phase 1	PD-1 blockade	10	China
Gastric cancer	NCT06405113	Phase 2	Sintilimab	198	China
Non-small cell lung cancer	NCT06403111	Phase 2	Tislelizumab	62	China
Metastatic colorectal cancer	NCT04729322	Phase 2	Pembrolizumab, Nivolumab	15	United States
Non-small cell lung cancer	NCT05008861	Phase 1	Pembrolizumab, Nivolumab, Durvalumab, Sintilimab, Tislelizumab, Camrelizumab	20	China
Advanced colorectal cancer	NCT06931808	Phase 4	Sintilimab	20	China
Renal cell carcinoma	NCT04758507	Phase 1, 2	ICB	50	Italy
Melanoma	NCT06623461	Phase 2	ICB	128	Canada
Liver cancer	NCT05750030	Phase 2	Atezolizumab	12	Austria

## Gut pathobionts and antitumor immunity

**Fig. 1:**
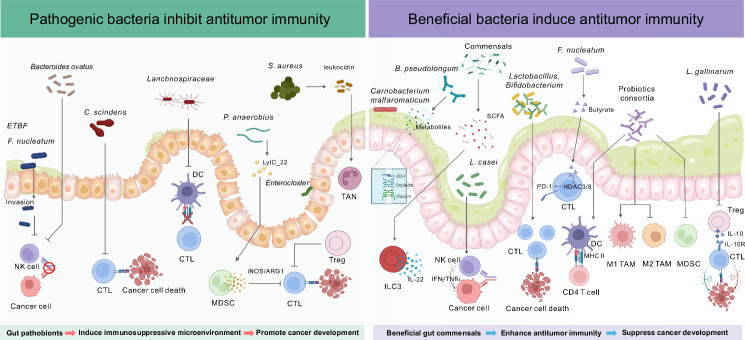
Gut microbiota modulation of antitumor immunity. The gut microbiota is closely associated with antitumor immunity, and gut pathobionts (e.g., ETBF, *P. anaerobius*, and *S. aureus*) promote immunosuppression to facilitate the escape of tumor cells from immunosurveillance. These pathogenic bacteria can (1) disrupt the gut barrier to induce inflammation and bacterial invasion; (2) suppress host antitumor immunity to favor tumor cell survival; (3) inhibit the function, differentiation, and maturation of antitumor immune cells (e.g., cytotoxic CD8^+^ T cells and NK cells) with reduced secretion of proinflammatory cytokines; and (4) increase the intratumoral infiltration of immunosuppressive immune cells (e.g., MDSCs and Tregs). In contrast, these protumorigenic and immunosuppressive events are counteracted by beneficial gut commensals (e.g., *C. maltaromaticum*, *A. mucinphila*, *Lactobacillus*, and *Bifidobacterium*). CTL cytotoxic T lymphocyte. Figure created with BioRender.com

The host immune system constitutes a critical defense against cancer development. However, such defense is actively reprogrammed by gut pathobionts, thereby shifting the tumor microenvironment toward an immunosuppressive state. These pathogenic microbes affect both innate and adaptive immunity by interacting with diverse immune cell populations and manipulating intricate immune-related signaling pathways, thereby subverting antitumor surveillance and accelerating cancer progression (Fig. 1).

### Modulation of the innate immune response

#### Gut barrier and antimicrobial signaling

The gut epithelial barrier serves as the initial defense against pathogenic microbes. However, its integrity is markedly disrupted in cancer, concomitant with gut microbial dysbiosis characterized by enriched pathogenic bacteria and reduced beneficial commensals. Disruption of the gut barrier can be driven by lifestyle factors such as a Western or high-fat diet and cigarette smoking [16, 17]. Certain pathogenic bacteria, such as enterotoxigenic *Bacteroides fragilis* (ETBF), also impair the gut barrier, which secretes virulence factors, particularly ETBF toxin, to damage the integrity of tight junctions, increase intestinal permeability, and induce systemic inflammation [18, 19]. Once the gut barrier is breached, a series of pattern recognition receptors (PRRs), including Toll-like receptors (TLRs), NOD-like receptors (NLRs), and RIG-I-like receptors (RLRs), are activated. These receptors recognize gut microbe-derived pathogen-associated molecular patterns (PAMPs) and host cell-derived damage-associated molecular patterns (DAMPs), which contain alarm signals owing to endogenous and exogenous damage caused by exposure to hydrophobic portions [20]. The activation of these receptors consequently triggers chronic inflammation, stimulating oncogenic signaling pathways such as cyclic GMP-AMP synthase (cGAS)-stimulator of interferon genes (STING) and nuclear factor kappa-light-chain-enhancer of activated B cells (NF-κB) [21].

Gut pathobionts also suppress the production of antimicrobial peptides (AMPs), such as defensins, bacteriocins, cathelicidins, and histatins, all of which are crucial for maintaining intestinal homeostasis. *Fusobacterium nucleatum* is a key bacterium that promotes and inhibits antitumor immunity in CRC, depending on the status of microsatellite instability (MSI). In particular, the abundance of *F. nucleatum* is positively correlated with microsatellite stable (MSS) tumors (approximately 85% of CRC cases), which are classified as immune “cold” tumors because of the lack of immune cells in the tumor microenvironment [22]. On the other hand, it is negatively correlated with MSI-high/deficient mismatch repair (dMMR) tumors (approximately 15% of CRCs), which are considered immune “hot” tumors [22]. In MSI-high/dMMR CRC, *F. nucleatum* induces DNA methyltransferase in host cells to epigenetically silence tumor-suppressing genes [23] and downregulate AMP expression in colon epithelial cells [24]. *F. nucleatum* also secretes several pathogenic proteins, such as the adhesin FadA, which promotes epithelial adhesion, and Fap2, which interacts with immune cells to induce immunosuppression [25]. Interestingly, increasing evidence has shown that CRC patients with a high abundance of *F. nucleatum* have a better response to PD-1 blockade immunotherapy than do those with a low abundance of *F. nucleatum*. Preclinical studies revealed that *F. nucleatum* enhances the antitumor immune response by converting immune “cold” tumors into “hot” tumors in mice, thereby increasing the efficacy of ICB [26, 27]. One possible mechanism is that *F. nucleatum* disrupts the function and integrity of the gut barrier, allowing systemic translocation of beneficial factors from its secretome, particularly butyrate [26]. Taken together, these findings indicate that gut microbial dysbiosis with enriched gut pathobionts can disrupt the gut barrier and inhibit antimicrobial signaling, collectively resulting in an environment conducive to tumor development.

#### Innate defense of myeloid cells

Myeloid cells, which refer mainly to granulocytes, monocytes, macrophages, and dendritic cells (DCs), originate from monocytes or neutrophils to mediate innate defense. The principal roles of myeloid cells in cancer include pathogen defense, inflammatory regulation, tissue homeostasis maintenance, and repair processes [28]. Gut pathobionts can modulate myeloid cells to facilitate tumorigenesis. A preclinical study by Thomas and colleagues demonstrated that both the gut microbiota and intrapancreatic microbiota impact the development of pancreatic ductal adenocarcinoma (PDAC) in mice, in which intrapancreatic microbes suppress the intratumoral infiltration of innate immune cells via TLR-mediated reprogramming of the tumor microenvironment [29]. Through fluorescence labeling, *Enterococcus faecalis* and *Escherichia coli* were found to migrate from the intestines into pancreatic tumor lesions, where they drive immunosuppression. PRRs activated by microbial stimuli in the tumor microenvironment, especially TLR2, TLR4, and TLR5, also enhance PDAC progression by amplifying both innate and adaptive immunosuppression. On the other hand, eradicating these intrapancreatic bacteria could induce tumor-associated macrophage (TAM) polarization toward “M1” proinflammatory phenotypes and reduce the number of CD206^+^ “M2”-like TAMs for cancer innate defense, thereby restoring antitumor immunity [30].

#### Natural killer cells

Natural killer (NK) cells exhibit innate cytotoxicity against tumor cells [31]. They are the primary producers of interferons (IFNs), TNF-α, granulocyte macrophage-colony stimulating factor (GM-CSF), and other immunomodulatory cytokines and chemokines, which collectively facilitate the recruitment and activation of hematopoietic cells through antigen-specific T-cell responses and bidirectional interactions with DCs and neutrophils. Upon tumor recognition, NK cells exocytose perforin-containing cytolytic granules to permeabilize cellular membranes and granzymes to induce programmed cell death [32]. However, the antitumor functions of NK cells are strongly disrupted by gut pathobionts, allowing tumors to more easily escape immune surveillance. In mice bearing PDAC or lung cancer, increased tumor growth was observed in mice with antibiotic-induced gut microbiota depletion, suggesting the critical role of gut microbes in regulating the NK cell-mediated antitumor response [33, 34]. Another study reported that *Helicobacter pylori* decreases the expression of activation receptors on NK cells, such as NKG2D, which is an important activator of mucosal immunity and tumor immunosurveillance in NK, cytotoxic, and γδ T cells [35]. This markedly inhibits the antitumor ability of NK cells, thereby facilitating the immune escape of tumor cells and promoting gastric cancer [36]. In hepatocellular carcinoma (HCC), enriched *Bacteroidetes ovatus* metabolizes chenodeoxycholic acid into isolithocholic acid to impair the cytotoxicity of hepatic NK cells in a phosphorylated CREB1-dependent manner, resulting in accelerated tumor progression [37].

#### Innate lymphoid cells

Innate lymphoid cells (ILCs), defined as innate immune effectors with adaptive-like functions, encompass three broad subsets: Group 1 ILCs (including ILC1s and NK cells), ILC2s, and ILC3s. These ILCs are capable of producing T helper (Th)1-type (e.g., IFN- γ), Th2-type (e.g., interleukin (IL)-5 and IL-13), and Th17/Th22 (e.g., IL-17 and IL-22)-type cytokines, respectively [38]. While these ILC subsets exhibit typical morphological traits of lymphocytes, they do not possess antigen-specific receptors [39]. In cancer, gut pathobionts can reprogram the activities of ILCs to foster a protumorigenic microenvironment. For example, patients with CRC with ILC3 dysfunction and chronic intestinal inflammation harbor an enrichment of *Bacteroides*, *Ruminococcaceae*, and Lachnospiraceae, which are pathologically associated with cancer immune evasion [40]. Other pathobionts, such as *E. coli, B. fragilis, Epsilon-Proteus*, and *gamma-Proteus*, secrete virulence factors, such as colicin, *B. fragilis* toxin, and cytolethal distending toxins (CDT), to decrease the expression of major histocompatibility complex (MHC)-II on ILC3s to undermine antitumor Th1 cell immunity [41, 42]. Hence, these findings highlight the intricate crosstalk between the gut microbiota and ILCs in shaping the tumor immune landscape.

### Modulation of the adaptive immune response

#### Cytotoxic CD8^+^ T cells

CD8^+^ T cells are key players in antitumor immunity, as they can directly kill tumor cells through perforin and granzyme secretion. However, gut pathobionts actively impair the cytotoxic function of CD8^+^ T cells to facilitate tumor immune escape. For example, gut microbe-derived deoxycholic acid (DCA) impairs CD8^+^ T-cell effector function by inhibiting plasma membrane Ca2^+^ ATPase and the downstream nuclear factor of activated T cells 2 (NFAT2) signaling pathway. Targeting DCA-producing *Clostridium scindens* by bacteriophages significantly reduces the expression of TNF-α, IFN-γ and granzyme B in intratumoral T cells, subsequently abolishing tumor growth in CRC [43]. Lattanzi and colleagues reported that the enrichment of *Parvimonas*, *Anaerococcus*, and *Alloprevotella* is associated with a reduction in CD8^+^ T cells and the tumor immunosuppressive response in an estrogen-dependent manner against CRC [44]. A recent study revealed that colibactin-producing *E. coli* suppresses the intratumoral infiltration of IFN-γ-producing CD8^+^ T cells in patients with right-sided CRC [45]. Moreover, patients colonized by colibactin-producing *E. coli* have significantly lower survival at stages III-IV of CRC than do CRC patients at the same stage without colonization. On the other hand, a mutant *E. coli* strain that does not produce colibactin (11G5∆ClbQ) fails to induce immune surveillance evasion and metabolic dysregulation in human CRC cells, confirming the immunomodulatory role of colibactin in CRC. Together, these studies offer insights into the crosstalk between CD8^+^ T cells and gut pathobionts in tumor immune evasion.

#### Antigen-presenting cells

Antigen-presenting cells (APCs), including TAMs and DCs, play crucial roles in triggering the adaptive immune response, whereas the antigen-presenting capacity of these cells can be altered by gut pathobionts to facilitate immune evasion. For example, ETBF suppresses DCs by increasing the expression of heme oxygenase-1 (HO-1), which is the key regulator of the DC-mediated anti-inflammatory response [46]. *H. pylori* upregulates MHC-II, CD80, CD86, and CD83 costimulatory molecules on DCs and stimulates IL-12 secretion to potentiate the maturation and activation of DCs. Moreover, *H. pylori* can induce IL-10 secretion in DCs to activate signal transducer and activator of transcription 3 (STAT3) signaling, simultaneously suppressing IL-1β secretion and impairing T-cell priming capacity [47]. On the other hand, butyrate derived from Lachnospiraceae species also inhibits IFNγ-secreting CD8^+^ T cells by restraining STING activation in DCs, causing CD8^+^ T-cell dysfunction [48]. Furthermore, *Bacteroides* mediate chemokine production by TAMs (e.g., CCL3), thereby recruiting CD8^+^ T cells to tumor niches and establishing a protumorigenic immune-stromal network in gliomas [49]. Overall, gut pathobionts can orchestrate the immunosuppressive TME by hijacking the functionality of APCs, primarily through TAM polarization and DC maturation, to reduce the cytotoxicity of CD8^+^ T cells.

#### Immunosuppressive myeloid cells

Myeloid-derived suppressor cells (MDSCs) represent a heterogeneous pool of immature myeloid cells that preferentially accumulate within the tumor microenvironment. These immunosuppressive cells inhibit the recruitment and activation of cytotoxic CD8^+^ T cells through multiple mechanisms, including the secretion of the immunosuppressive cytokines IL-10 and transforming growth factor-β (TGF-β), as well as the enzymatic depletion of essential amino acids required for T-cell activity. Notably, the proliferation and immunosuppressive function of MDSCs can be amplified by gut pathobionts. *Peptostreptococcus anaerobius* is an oral pathogen that modulates the tumor immune microenvironment to facilitate CRC development. On the one hand, *P. anaerobius* engages integrin α2β1 signaling on tumor cells to activate NF-κB and stimulate CXCL1 production, thereby promoting MDSC chemotaxis into tumors [50]. On the other hand, *P. anaerobius* generates the functional component LytC_22, which induces the immunosuppressive programming of MDSCs via Slamf4 receptor ligation. Moreover, blockade of the integrin α2β1 or Slamf4 receptor reversed immune evasion induced by *P. anaerobius*. Another study reported that the gut microbiota and the metabolite taurocholic acid (TCA) contribute to lung metastasis by triggering MDSC accumulation in mice bearing CRC tumors [51]. TCA further promotes glycolysis in MDSCs by epigenetically enhancing the monomethylation of H3K4 in target genes and inhibiting CHIP-mediated ubiquitination of PD-L1. Moreover, *Helicobacter* can drive the polarization of Schlafen (SLFN4)-expressing MDSCs in the gastric mucosa, and the proportion of SLFN4^+^ MDSCs is associated with an increased incidence of intestinal metaplasia and gastric tumorigenesis [52].

In addition to bacteria, an impaired gut barrier caused by enriched gram-negative gut pathobionts also induces the accumulation of polymorphonuclear (PMN)-MDSCs in the liver via the lipopolysaccharide (LPS)/TLR4 axis, subsequently promoting cholangiocarcinoma [53]. In addition, high-fat diet-induced gut microbial dysbiosis with increased *Desulfovibrio* abundance is strongly correlated with worse prognosis and advanced clinicopathological status in patients with breast cancer, in which enriched pathobionts release excess leucine to promote tumor progression by generating PMN-MDSCs [54].

In addition to MDSCs, tumor-associated neutrophils (TANs) are another large immune cell population with immunosuppressive functions that are recruited to the tumor microenvironment. In particular, TANs can produce various reactive oxygen species (ROS) and other immunosuppressive mediators to inhibit cytotoxic CD8^+^ T-cell activation. Moreover, gut microbial dysbiosis also drives peritoneal metastasis in CRC by recruiting TANs to disrupt stromal integrity and enhance the invasiveness of tumor cells, whereas therapeutic targeting of this TAN–microbe crosstalk can stabilize the tumor microenvironment and counteract metastasis [55]. Bhattacharya and colleagues reported that *Staphylococcus aureus* secretes Panton-Valentine leukocidin, a powerful inducer of neutrophil extracellular traps (NETs) that specifically target the mitochondrial membrane of TANs, thereby triggering ROS overproduction and eventually driving CRC metastasis in mice [56]. Moreover, neutrophil elastase is another major mediator of the formation of NETs. Interestingly, a murine study revealed that *Porphyromonas gingivalis* can increase the secretion of neutrophil elastase by TANs to promote PDAC progression; however, the underlying mechanism is undetermined [57].

#### Immunosuppressive T cells

In addition to CD8^+^ T cells, the gut microbiota also plays a role in maintaining the homeostatic function of regulatory T (Treg) cells and IL-17-producing Th17 cells [58], whereas gut pathobionts can drive the differentiation of these immunosuppressive T-cell subsets to suppress antitumor immunity. A murine study revealed that antibiotic treatment results in a reduction in MAdCAM-1 in ileal venules, which is attributed to the recolonization of the gut *enterocloster*, especially in the species *clostridioformis* [59]. This change coincides with the migration of Th17 cells from the ileum to extraintestinal tumors and to tumor-draining lymph nodes in cancer. In another study, treatment with broad-spectrum antibiotics led to an over 90% reduction in the number of gut pathobionts in mice, which in turn attenuated HCC development [60]. These antitumor effects are accompanied by a decrease in liver and circulating immunoglobulin (Ig)-A levels, as well as Th17 cells. Moreover, ETBF can induce the differentiation of Th17 cells, thereby contributing to the proliferation of CRC cells [61]. For Tregs, *F. nucleatum* establishes self-reinforcing colonization in esophageal squamous cell carcinoma by enriching immunosuppressive Tregs, simultaneously dampening antitumor immunity and accelerating malignant progression [62]. In addition, *H. pylori* promotes gastritis and gastric tumorigenesis by inciting the local proliferation of CD4^+^CD25^hi^ forkhead box protein 3 (FoxP3^+^) Tregs [63] and/or disrupting the balance between Th1 and Th17 cells to sustain chronic inflammation [64].

#### B cells

In the context of antitumor immunity, B cells perform two-sided functions, demonstrating both protumorigenic and antitumorigenic capacities. Moreover, gut pathobionts can modulate B-cell-mediated immunity through altering antibody production and cytokine release. For example, *H. pylori*, together with its effector CagA, induces immunosuppressive IgA production and Th17 cell generation [65]. Infants born to mothers suffering from IBD have reduced microbial diversity, particularly marked by *Bifidobacterium* depletion, which impairs memory B-cell class-switching during early immune development [66]. Nonetheless, more studies are needed to illustrate the pivotal role of gut pathobionts in orchestrating B-cell-dependent antitumor immunity.

## Beneficial bacteria and/or probiotics in antitumor immunity

In addition to gut pathogens, interest in the roles of beneficial bacteria and/or probiotics in modulating the immune system as a strategy against cancer is increasing. Emerging evidence has shown that these beneficial microbes enhance antitumor immunity by affecting both innate and adaptive immune responses. In this section, the current understanding of how beneficial bacteria and/or probiotics interact with the immune system to suppress cancer is explored (Fig. 1).

### Innate immune response

#### Gut barrier function and antimicrobial signaling

Probiotics play a vital role in maintaining the function and integrity of the gut barrier. Several studies have demonstrated the beneficial effects of probiotics on the gut barrier in CRC mouse models. These probiotics include *Carnobacterium maltaromaticum* and *Roseburia intestinalis*, which improve gut barrier integrity by upregulating tight junction proteins (e.g., claudins, occludin, and zonula occludens (ZO)-1) while lowering serum LPS levels to suppress colorectal tumorigenesis in mice [67, 68]. In mice with metabolic dysfunction-associated steatotic liver disease-associated HCC, *Bifidobacterium pseudolongum* restores the gut microbiota and improves gut barrier function by generating the metabolite acetate [69]. Another study revealed that *B. dentium* activates autophagy and calcium signaling to stimulate the release of mucus from goblet cells through gamma-aminobutyric acid (GABA) production [70]. In general, an improved gut barrier is more effective at restricting pathogenic bacterial translocation and the dissemination of associated toxins, thereby mitigating inflammation and tumorigenesis. Moreover, probiotics also regulate antimicrobial signaling. For example, *Bifidobacterium longum* subsp. *infantis YLGB-1496* increases the expression of AMP genes, including cathelicidin protein (CAMP), human β-defensin (hBD)-2, and hBD-3, in HaCaT and reconstituted human epidermis cells [71]. This effectively inhibits the adhesion of pathogenic bacteria and consequently alleviates intestinal inflammation.

#### Myeloid cells

Several probiotics suppress tumor progression by acting on the innate defense mechanism of myeloid cells. For example, *Lactobacillus casei* enhances antitumor immunity by increasing the phagocytic activity of macrophages and stimulating their polarization toward the “M1” phenotype with increased secretion of proinflammatory cytokines (e.g., TNF-α and IL-1β) [72]. *Lactobacillus salivarius* triggers the production of proinflammatory cytokines and upregulates the costimulatory molecules CD80 and CD86 on DCs, thereby promoting their moderate maturation and activation for innate defense [73].

#### Natural killer cells

Probiotics can increase the immunosurveillance efficiency of NK cells by increasing their capacity to eradicate tumor cells. An increasing number of studies have suggested that *L. casei Shirota* augments NK cell cytotoxicity and preferentially induces the expression of CD69 and CD25 on CD8^+^ and CD56^+^ NK subsets. Treatment with these bacteria significantly reduces the recurrence of superficial bladder cancer [74] and CRC with moderate atypia in patients [75]. Clark and colleagues reported that *Streptococcus pneumoniae* increases IL-10 production from NK cells in the lung and restricts host defense [76] (IL-10 is a well-known anti-inflammatory cytokine and tumor suppressor that eradicates tumor cells and enhances antitumor immune surveillance [77]). Moreover, the abundance of *S. pneumoniae* is lower in the lungs of mice with IL-10 deficiency than in those of wild-type controls. Functionally, the virulence protein Spr1875 of *S. pneumoniae* induces IL-10 production by NK cells. In addition, probiotics can upregulate IFN-γ and TNF-α in NK cells to mediate the differentiation of oral squamous cancer stem cells, subsequently suppressing tumor development while attenuating proinflammatory cytokine cascades [78].

#### Innate lymphoid cells

The function of ILCs is mediated by probiotics, which can improve antitumor immunity in the tumor microenvironment. For example, the administration of extracellular vesicles derived from *Lactobacillus reuteri EHA2* significantly enhances host immunity by reducing the population of IFN-γ^+^ ILC1s in both pulmonary and small intestinal lesions [79]. ILC2s are a subset that exhibits protumorigenic activity. Mechanistic studies reported that butyrate derived from the gut microbiota inhibits IL-13/IL-5 secretion from ILC2s via histone deacetylase (HDAC)-dependent epigenetic modulation in humans and mice, leading to a favorable immune microenvironment for tumor control [80]. ILC3s represent the primary lymphoid subset that directly interacts with the gut microbiota. Notably, probiotics can enhance ILC3 effector function and promote IL-22 production to increase AMP secretion and mucin biosynthesis by intestinal epithelial cells. Through FMT, recipient mice transplanted with a microbiota enriched with *Akkermansia* demonstrated potent anti-inflammatory and protective effects on the integrity of the gut mucosal barrier by orchestrating RORγt^+^ ILC3s in mucosal immunity [81]. In mice with HCC, *L. reuteri* administration elevates the intestinal acetate concentration to downregulate IL-17A in hepatic ILC3s through HDAC inhibition, ultimately attenuating neoplastic progression [82]. Similarly, Yang and colleagues reported that short-chain fatty acids (SCFAs) produced by the gut microbiota enhance IL-22 production in ILC3s via HDAC inhibition and G protein-coupled receptor (GPR)-41 signaling to abolish intestinal inflammation [83].

### Adaptive immune response

#### Cytotoxic CD8^+^ T cells

Numerous studies have reported the critical roles of the gut microbiota in modulating cytotoxic CD8⁺ T-cell function and adaptive antitumor immunity. Using germ-free mice, Tanoue and colleagues identified a consortium of 11 human-derived gut commensal strains that are capable of inducing IFN-γ⁺ CD8⁺ T cells to potentiate anticancer immunity [84]. Wang and colleagues demonstrated that a purified membrane protein from *Akkermansia muciniphila* can expand the intestinal CD8⁺ T-cell population, thereby inhibiting CRC tumorigenesis in carcinogen-treated mice [85]. Similarly, *Bifidobacterium breve JCM92* supplementation also inhibits CRC development through CD8⁺ T-cell expansion [86].

To elucidate the underlying mechanisms, recent studies have focused on gut microbe-driven epigenetic regulation of CD8⁺ T cells via microbial metabolites such as SCFAs and indole derivatives. In a preclinical study in 2024, *F. nucleatum* was shown to produce butyrate to decrease PD-1 expression and reactivate CD8^+^ T cells in humanized mouse models and in mice bearing MSS CRC tumors [26]. Mechanistically, *F. nucleatum*-derived butyrate downregulates the activity of HDAC3 or HDAC8 to selectively target H3K27ac and modulate PD-1 expression, alleviating the exhaustion of intratumoral CD8⁺ T cells. These effects were confirmed via the use of a bioengineered *F. nucleatum* strain with a mutant enoyl-CoA hydratase (an essential bacterial enzyme for butyrate fermentation), which loses immunomodulatory function with no impact on antitumor immunity. Jia and colleagues revealed that *Lactobacillus johnsonii* synergizes with *Clostridium sporogenes* to produce indole-3-propionic acid (IPA) [87]. IPA then reprograms CD8⁺ T-cell stemness by promoting H3K27 acetylation at Tcf7 superenhancers to facilitate the generation of progenitor exhausted CD8^+^ T cells, thereby augmenting the antitumor immune response against melanoma, breast cancer, and colorectal cancer. In line with these findings, IPA produced by *Lactobacillus plantarum L168* is capable of alleviating intestinal inflammation and tumor growth, which inhibits Saa3-linked cholesterol metabolism in CD8⁺ T cells through chromatin remodeling to increase the cytotoxic function of tumor-infiltrating CD8⁺ T cells in CRC [88]. In addition to indole derivatives, microbe-derived SCFAs also directly enhance the antitumor capacity of CD8⁺ T cells by inducing the effector function, metabolic fitness, and memory potential of antigen-activated CD8^+^ T cells [89, 90]. Specifically, pentanoate and butyrate derived from gut commensals such as *Megasphaera massiliensis* inhibit HDAC class I enzymes while activating mTOR in CD8⁺ T cells to increase their antitumor function in mice with PDAC [91]. *R. intestinalis*-derived butyrate also suppresses CRC development by activating granzyme B⁺, IFN-γ⁺, and TNF-α⁺ CD8⁺ T cells in mice [68].

#### Antigen-presenting cells

APCs constitute key targets for probiotic-mediated antitumor immunity. For example, gut microbes ferment dietary fibers to generate SCFAs that activate GPR109A signaling [92]. GPR109A activation then confers anti-inflammatory effects on colonic macrophages and DCs, leading to the induction of Treg and IL-10-producing T cells to suppress CRC development. Han and colleagues established a smectite-enhanced probiotic formulation that potently activated DC-mediated antitumor immunity and enhanced the adaptive immune response [93]. Moreover, multiple probiotic strains, including *Streptococcus thermophilus, B. breve Bb99, Lactococcus lactis subsp. cremoris, L. casei*, and *Bifidobacterium animalis*, also upregulate MHC-II and costimulatory molecules on human monocyte-derived DCs, although the cytokine secretion induced by these probiotics appears to be strain-specific [94, 95].

With respect to TAMs, probiotics enhance antigen presentation by promoting polarization toward the “M1” phenotype, thereby increasing their T-cell priming ability. A study reported that the combination of *L. casei* and *L. reuteri* induces M1 polarization via TLR4 signaling blockade to suppress PDAC progression [96]. Notably, *A. muciniphila*-derived extracellular vesicles can reprogram macrophage polarization by increasing the number of tumor-killing M1 macrophages while decreasing the immunosuppressive M2 population in mice with PDAC [97]. Additionally, *B. breve* treatment markedly ameliorates colitis and colitis-associated tumorigenesis through tryptophan metabolic pathway modulation [98].

#### Immunosuppressive cells

Probiotics antagonize the immunosuppressive function of Tregs and modulate T-cell-mediated adaptive immunity. For example, *Prevotellaceae*, *Rikenella*, and *Fournierella* contribute to the maintenance of intestinal homeostasis during intestinal disorders [99]. Notably, a clinical and murine study revealed that microbial signatures correlated with increased levels of tumor-infiltrating T-cell subsets (e.g., Th1, Treg, T follicular helper, and Th17), which are also associated with improved survival [100]. Through FMT, the transplantation of probiotic-enriched microbiota increases the number of CD4^+^CD25^+^Foxp3^+^ Tregs in recipient mice and subsequently suppresses colitis-associated tumorigenesis [101]. At the species level, Fong and colleagues demonstrated that *Lactobacillus gallinarum* supplementation reduces the intratumoural infiltration of Tregs while increasing CD8^+^ T-cell effector function in mice with CRC [102]. Mechanistically, *L. gallinarum* generates indole-3-carboxylic acid to competitively inhibit the binding of protumorigenic kynurenine to the aryl hydrocarbon receptor (AHR) on CD4^+^ T cells, thereby inhibiting Treg differentiation.

Compared with research on T cells, current research regarding the ability of probiotics to counteract immunosuppressive MDSCs and TANs is lacking. A previous study reported that the gut microbiota is altered in germ-free mice transferred to standard housing conditions postweaning, concomitant with upregulation of proinflammatory CXCL1/2/5 and accumulation of granulocytic MDSCs [103]. These transferred mice have increased susceptibility to colitis-associated colorectal tumorigenesis in adulthood. Bacterial supplementation also increases peripheral neutrophil counts in germ-free mice, while TANs in small or early-stage tumors exhibit robust tumor-killing activity through the production of cytotoxic TNF-α and IFN-γ [104]. These findings highlight the crucial roles of early-life microbial or probiotic exposure in establishing intestinal homeostasis, which confers lifelong protection against malignancy. Further mechanistic studies of the crosstalk between immunosuppressive cells and the gut microbiota in cancer development are warranted.

## The gut microbiota and cancer immunotherapy

**Fig. 2:**
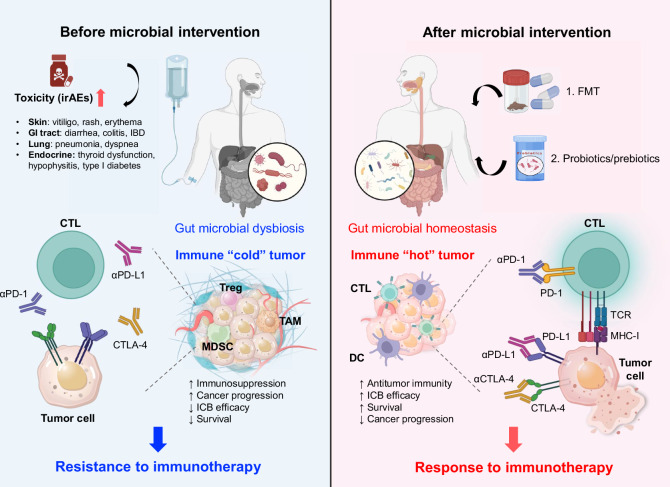
Gut microbiota modulation in cancer immunotherapy. The gut microbiota influences not only the efficacy of cancer immunotherapy but also the occurrence of irAEs. Patients resistant to immunotherapy generally have a dysbiotic gut microbiota and so-called immune “cold” tumors, which have low infiltration of cytotoxic T lymphocytes (CTLs), resulting in immunosuppression, reduced treatment efficacy and survival, and irAEs. Notably, such therapeutic resistance can be alleviated by microbial interventions, including FMT, probiotics, and prebiotics. These strategies can restore the gut microbiota composition while potentiating the intratumoral infiltration of effector CD8^+^ T cells to target and kill tumor cells. Eventually, the efficacy of cancer immunotherapy is improved by the synergistic use of microbial treatment. TCR. T-cell receptor. Figure created with BioRender.com

Immunotherapy has emerged as a revolutionary breakthrough in cancer treatment since the Nobel Prize-winning discovery of immune checkpoint blockade (ICB) in 2018. In addition to ICB, chimeric antigen receptor (CAR)-T-cell therapy and CAR-NK-cell therapy have also achieved remarkable clinical success across diverse malignancies. Nevertheless, the response rate to immunotherapy remains heterogeneous, prompting investigations to develop strategies for improving its efficacy. Recent evidence has demonstrated that the gut microbiota is a key regulator of the tumor microenvironment, thus profoundly shaping immunotherapy outcomes (Table 2). In this section, we delve into the current understanding of how the gut microbiota affects the efficacy of ICB and CAR-T-cell immunotherapy (Fig. 2).

**Table 2:** Clinical trials of probiotics as adjuvants in cancer immunotherapy

Cancer type	Study number	Phase	Probiotics	Immunotherapy	Enrollment	Country
Advanced urothelial carcinoma	NCT06904573	Phase 2	Biolosion (*Lactobacillus rhamnosus, Lactobacillus paracasei*, *Bifidobacterium lactis*, *Lactobacillus acidophilus*)	Pembrolizumab, Toripalimab	222	China
Bladder urothelial carcinoma	NCT05220124	Phase 4	*Bifidobacterium, Lactobacillus, Enterococcus*	ICB	190	China
Liver cancer	NCT05032014	N/A	*Lactobacillus rhamnosus Probio-M9*	ICB	46	China
Non-small cell lung cancer	NCT04699721	Phase 2	*Bifidobacterium trifidum*	Nivolumab	60	China
Triple negative breast cancer	NCT06768931	Phase 2	Biolosion (*Lactobacillus rhamnosus, Lactobacillus paracasei*, *Bifidobacterium lactis*, *Lactobacillus acidophilus*)	PD-1 blockade	192	China
Metastatic non-small cell lung cancer	NCT06428422	N/A	*Bifidobacterium animalis subsp. lactis Bl-04*	Nivolumab	100	Turkey
Anal canal squamous cell cancer	NCT03870607	Phase 2	Symbioflor® 1 *(Enterococcus faecalis*)	ICB	75	Brazil
Advanced esophageal squamous cell carcinoma	NCT06401447	N/A	*Clostridium Butyricum MIYAIRI 588*	Sintilimab	50	China
Non-small cell lung cancer	NCT05094167	N/A	*Lactobacillus Bifidobacterium V9* (*Lactobacillus rhamnosus*)	ICB	46	China
Renal cell carcinoma	NCT03829111	Phase 1	*Clostridium butyricum CBM 588*	Ipilimumab, Nivolumab	30	United States
Renal cell carcinoma	NCT05122546	Phase 1	*Clostridium butyricum CBM 588*	Nivolumab	31	United States
Metastatic colorectal cancer	NCT06823323	N/A	*Lactobacillus johnsonii*	Pembrolizumab	150	China

### Modulation of the CD8^+^ T-cell response in the ICB

The therapeutic efficacy of most cancer immunotherapies fundamentally relies on the effector function of cytotoxic CD8^+^ T cells. Multiple retrospective studies have reported that antibiotic use is correlated with reduced survival and a blunted response to ICB in patients with advanced solid tumors, implying that antibiotics may impair ICB efficacy by inducing gut microbial dysbiosis [105–109]. Other studies revealed that the gut microbiota exerts substantial effects on the CD8^+^ T-cell response during ICB. Kim and colleagues demonstrated that transferring stool from anti-PD-1 responders could restore therapeutic sensitivity to anti-PD-1 inhibitors in recipient patients with refractory solid cancer, with an objective response rate of 7.7% [110]. FMT also increases CD8^+^ T-cell infiltration and activation in both the tumor microenvironment and the host immune system. Moreover, microbial species can induce CD8^+^ T-cell activation in the peripheral blood and tumor niches. For example, melanoma patients with enriched Clostridiales, Ruminococcaceae, and *Faecalibacterium* have better effector functions of intratumoral and peripheral CD8^+^ T cells, thereby potentiating ICB efficacy [111].

The mechanisms underlying the gut microbe-mediated modulation of the CD8^+^ T-cell response during ICB are multifaceted. A primary mechanism centers on SCFA production, which enhances CD8^+^ T-cell differentiation and effector function. For example, butyrate-producing *R. intestinalis* suppresses tumor growth by expanding cytotoxic granzyme B^+^, IFN-γ^+^ and TNF-α^+^ CD8^+^ T cells, concurrently enhancing anti-PD-1 efficacy in mice with MSS CRC [68]. In general, MSS CRC accounts for 85% of all CRC cases, yet the efficacy of ICB for treating MSS CRC remains markedly unsatisfactory due to the lack of tumor-infiltrating immune cells in this subtype. Interestingly, Wang and colleagues reported that the abundance of *F. nucleatum* is negatively correlated with PD-1 expression in patients with MSS CRC [26]. The transfer of stool from *F. nucleatum*-enriched MSS-CRC patients confers anti-PD-1 sensitivity in recipient germ-free mice, highlighting the beneficial effects of *F. nucleatum* in the immune checkpoint blockade (ICB) against MSS CRC. Mechanistically, *F. nucleatum*-derived butyrate reduces CD8^+^ T-cell exhaustion, subsequently increasing its effector function via the HDAC3/8-TBX21 axis to overcome ICB resistance. A secondary mechanism involves human leukocyte antigen (HLA)-mediated antigen presentation. As the central immune recognition complex, the HLA directs antigenic peptide presentation to CD8^+^ T cells. Moreover, combining FMT with PD-1 blockade leads to the upregulation of HLA class II genes (CD74, GZMK) on tumor-infiltrating CD8^+^ T cells while activating CD56^+^CD8^+^ T-cell subsets [11]. These findings collectively show that modulating the gut microbiota is a potent strategy to amplify CD8^+^ T-cell-driven antitumor immunity during ICB.

### Modulation of Th1 and Th17 responses in the ICB

Th1 and Th17 cells represent two functionally distinct T helper cell subsets. Th1 cells combat intracellular pathogens through IFN-γ/TNF-α secretion, macrophage activation, and cell-mediated immunity amplification. Th17 cells counter extracellular threats mainly via IL-17/IL-21/IL-22 production, driving inflammation and neutrophil recruitment. While both subsets are immunoprotective, a dysregulated Th1 response is associated with autoimmune disorders, whereas Th17 imbalance contributes to inflammation. The gut microbiota actively shapes the Th1/Th17 response during ICB. Gopalakrishnan and colleagues demonstrated that transferring stool from anti-PD-1 nonresponders expands the number of tumor-infiltrating RORγt^+^ Th17 cells and splenic CD4^+^IL-17^+^ Tregs in recipient mice, indicating that the gut microbiota drives immunosuppression in a Th17-dependent manner [111]. On the other hand, *B. fragilis* restores ICB efficacy by promoting the IL-12-dependent Th1 response through the activation of lamina propria DCs [6]. Additionally, *B. pseudolongum* produces inosine to enhance Th1 differentiation and antitumor immunity, thereby potentiating ICI efficacy [112]. Hence, these findings indicate that Th1 and Th17 cells, in addition to CD8 + T cells, are robust targets for improving ICB efficacy.

### Modulation of innate immune cells in the ICB

The gut microbiota orchestrates the dynamics of innate immune cells to modulate ICB efficacy. For example, combining anti-PD-1 therapy with TREM2-deficient macrophages can reprogram intestinal macrophages toward proinflammatory phenotypes, accompanied by the enrichment of *Ruminococcus gnavus* in mice [113]. Notably, *R. gnavus* supplementation potentiates anti-PD-1-mediated tumor elimination in mice by inducing a proinflammatory intestinal microenvironment, which enhances the proliferation and infiltration of TNF-producing CD4^+^ T cells into tumors. In patients with NSCLC, gut microbial diversity is positively correlated with peripheral memory CD8^+^ T-cell and NK cell subsets following PD-1 blockade [114]. For NK cells, *L. plantarum* administration upregulates the natural cytotoxic receptor protein of NK cells, which in turn promotes NK cell activation and triggers the innate immune response [34]. Moreover, gut microbe-primed DCs exhibit increased CD8^+^ T-cell priming capacity during ICB, while microbiota profiling revealed that this effect, together with increased antitumor efficacy, is correlated with *Bifidobacterium* [115]. Indeed, *Bifidobacterium* administration restores the efficacy of PD-L1 blockade by promoting the maturation and infiltration of DCs in tumor niches and increasing their ability to prime for CD8^+^ T-cell activation.

### Modulation through host‒microbe interactions

Host‒microbe crosstalk results in the formation of a complex interaction network that regulates signaling pathways in the immune checkpoint complex (ICB), which plays dual immunomodulatory roles. In particular, the gut microbiota regulates immune checkpoint molecules (e.g., PD-1 and CTLA-4) through direct and indirect metabolic pathways, thereby modulating host immune responses against cancer. For example, microbe-derived tryptophan metabolites (e.g., kynurenine) regulate Treg differentiation via the AHR‒Treg axis [116]. These metabolites induce Foxp3 expression to promote Treg development while inhibiting RORγt-mediated Th17 cell differentiation. Specifically, kynurenine enhances Treg polarization through AHR activation to maintain immune homeostasis [117–119]. On the other hand, the expression of PD-1 in CD8^+^ T cells is upregulated by kynurenine, which interacts with AHR to induce T-cell exhaustion and reduce ICB efficacy [120]. Notably, *Enterococcus*-secreted SagA, a conserved NlpC/p60 peptidoglycan hydrolase, upregulates the expression of the innate immune sensor protein NOD2 to increase the ICB-induced antitumor response [121]. Similarly, polysaccharides from *Leuconostoc mesenteroides NTM048* or *B. fragilis* exert immunomodulatory effects by strengthening gut mucosal integrity and modulating systemic immune responses [122]. In addition, *Lactobacillus delbrueckii*-derived prostate-specific antigens (PSAs) increase ICB efficacy by eliciting CCR6^+^CD8^+^ T cells in patients and mice [123], suggesting that the host‒microbe interaction is a pivotal target for improving ICB efficacy.

### CAR-T-cell therapy and CAR-NK-cell therapy

CAR-T-cell therapy and CAR-NK-cell therapy represent advanced immunotherapeutic strategies that genetically engineer immune cells to express chimeric antigen receptors (CARs), enabling the precise targeting and killing of tumor cells. However, while CAR-T-cell therapy has shown remarkable efficacy against hematologic malignancies, its efficacy is greatly reduced when it is used to treat solid tumors. More importantly, CAR-T-cell therapy is associated with severe adverse effects, such as cytokine release syndrome (CRS) and immune effector cell-associated neurotoxicity syndrome, thus limiting its therapeutic success. In contrast, CAR-NK therapy is a safer alternative with lower risks of CRS and graft-versus-host disease [124]. The gut microbiota can modulate the efficacy of both CAR-T- and CAR-NK-cell therapies. Smith and colleagues established a cohort of CAR-T recipients who received either antibiotics or broad-spectrum agents (piperacillin/tazobactam, imipenem/cilastatin, meropenem (PIM)) to target anaerobic commensals, and their analysis revealed that PIM exposure prior to CAR-T-cell therapy was associated with significantly worse overall survival (OS) and progression-free survival (PFS) [125]. A recent study by Hu and colleagues explored the correlation between CAR-T-cell toxicity and changes in the gut microbiota in patients with relapsed/refractory multiple myeloma or non-Hodgkin lymphoma [126]. The results revealed that severe CRS is associated with reduced *Bifidobacterium*, whereas decreased alpha diversity after CAR-T-cell infusion is correlated with increased *Actinomyces* and *Enterococcus*. Moreover, patients who achieve a complete response have greater abundances of *Prevotella, Collinsella, Bifidobacterium*, and *Sutterella* than do those who achieve a partial response [126]. In addition to bacteria, microbial metabolites also mediate CAR-T-cell efficacy. A recent preclinical study reported that receptor tyrosine kinase-like orphan 1 (ROR1)-targeted CAR-T cells reduce the pancreatic tumor burden in mice, whereas treatment efficacy is increased by supplementation with microbe-derived butyrate and pentanoate [127].

## Gut microbiota-targeting strategies to increase the effectiveness of cancer immunotherapy

**Fig. 3:**
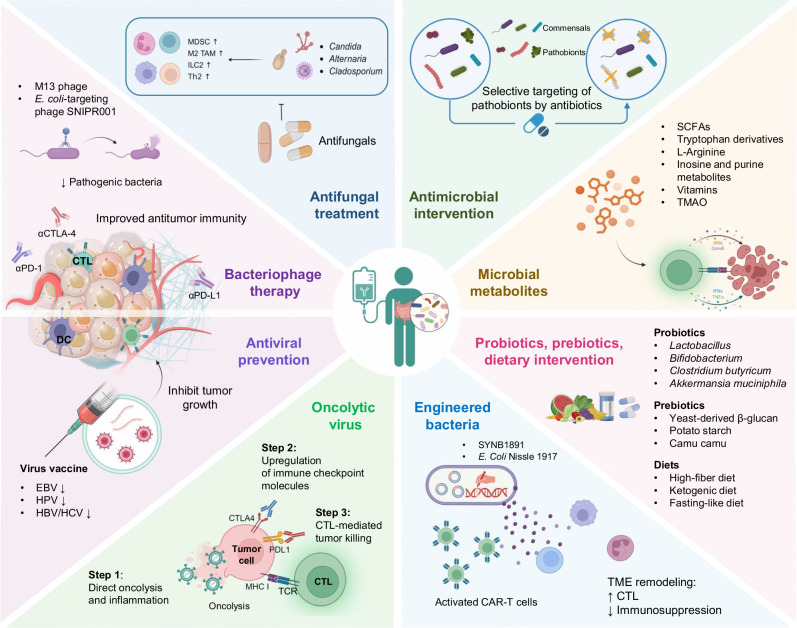
Gut microbiota-targeting strategies to boost cancer immunotherapy. There are different approaches that target the gut microbiota to improve the immunotherapy response. (1) Antimicrobial intervention, which uses antibiotics to selectively eradicate gut pathobionts without harming beneficial commensals. (2) Microbial metabolites (e.g., SCFAs, tryptophan derivatives, L-arginine, vitamins, inosine, TMAO), and (3) probiotics, prebiotics, and dietary intervention can activate cytotoxic T lymphocytes (CTLs) to potentiate antitumor immunity. (4) Engineered bacteria develop a bacterial strain to specifically target tumor cells, modulate the host antitumor response, and remodel the tumor microenvironment (TME). (5) Oncolytic viruses utilize engineered viruses to specifically target and destroy tumor cells through direct oncolysis and activation of CTLs and other antitumor immune cells. (6) Antiviral prevention, which utilizes vaccines targeting viruses, including EBV, HPV, HBV and HCV, to abolish their ability to infect tumor cells and elicit a protumorigenic immune response. (7) Bacteriophage therapy, which targets and directly kills gut pathobionts and detrimental bacteria to facilitate antitumor immune responses involving DCs and CTLs. (8) Antifungal treatments deplete protumorigenic fungi such as *Candida*, *Alternaria*, and *Cladosporium* and reduce the number of tumor-promoting immunosuppressive cells (e.g., MDSCs, TAMs, IL2, and Th2). TCR, T-cell receptor. Figure created with BioRender.com

Recent breakthroughs in the intricate interplay between host immunity and the gut microbiota have revealed novel approaches to improve immunotherapy efficacy. Here, we explore microbiota-targeted strategies with promising potential to augment antitumor immunity: (1) antimicrobial intervention, (2) microbial-derived metabolites, (3) prebiotic supplementation and dietary modulation, (4) genetically modified bacterial strains, (5) oncolytic virus immunotherapy, and (6) bacteriophage-targeted pathobiont therapy (Fig. 3).

### Antimicrobial intervention

Antimicrobial interventions, including antibiotics, antifungal agents, and precision-targeted inhibitors, exhibit dual effects in cancer treatment. While antimicrobial intervention is effective in preventing and treating cancer treatment-associated infections, broad-spectrum antibiotics can markedly disrupt the gut microbiota, potentially undermining immunotherapy efficacy. In particular, the use of broad-spectrum antibiotics during or before ICB is correlated with reduced PFS and overall survival in cancer patients. A gastric cancer study reported that pretreatment antibiotic use significantly diminishes PFS and overall survival in patients receiving anti-PD-1 therapy [128]. Mechanistically, antibiotics disrupt gut microbial diversity, reduce beneficial commensals such as *L. gasseri*, and drive the aberrant expansion of circulating exhausted CD8^+^ T cells. A systematic review of 107 studies (123 cohorts, 41,663 patients) revealed that 28% of cancer patients received antibiotics during ICB, while the analysis revealed that these patients had significantly worse clinical outcomes than those without antibiotic use [129]. Hence, this systematic review demonstrated that antibiotic use is significantly associated with poorer prognosis and worse survival in patients receiving ICB.

Emerging evidence has emphasized the critical need for pathogen-selective approaches that eradicate detrimental species while preserving beneficial gut commensals. Wang and colleagues developed a liposomal antibiotic delivery system that specifically targeted and eradicated *F. nucleatum* in CRC mouse models, which markedly increased the efficacy of ICB via microbial epitope exposure [130]. Similarly, another study developed *F. nucleatum*-mimetic nanocomplexes by encapsulating colistin in liposomes [131]. These colistin-loaded liposomes can fuse with the *F. nucleatum* cytoplasmic membrane to selectively kill intratumoral *F. nucleatum*-toxic strains without affecting other microbes, thereby synergizing with ICB to treat CRC.

Gut fungi are critically involved in various cancer types [132, 133]. For example, *Candida* species are enriched in CRC and HCC, where they enhance the immunosuppressive function of MDSCs, TAMs, and ILC2s to promote tumorigenesis [134–138]. The intratumoral infiltration of ILC2s and Th2 cells can also be induced by *Alternaria* species, thereby contributing to PDAC development [139]. *Cladosporium* species are associated with T-cell-related immune evasion in esophageal cancer, whereas targeting and eradicating these protumorigenic fungi is effective in increasing ICB efficacy [140]. Indeed, a recent study reported that rapamycin, an antifungal antibiotic produced by *Streptomyces hygroscopicus NRRL 5491* [141], enhances the anti-PD-1 response and simultaneously inhibits tumorigenesis, recurrence, and liver metastasis in CRC mouse models [142]. Mechanistically, rapamycin reshaped the immunosuppressive TME by depleting α-SMA^+^ cancer-associated fibroblasts, degrading collagens, increasing the intratumoral infiltration of T cells, and decreasing PD-L1 expression in tumor cells.

In addition to bacteria and fungi, viruses such as EBV, HPV, HBV and HCV also have the capacity to create a tumor microenvironment favorable for immune evasion and cancer development [143–148]. Studies are now investigating the potential of depleting protumorigenic viruses via prophylactic vaccines to mitigate the risk of virus-associated cancer [149]. In particular, a phase I clinical trial of patients with EBV-positive nasopharyngeal cancer utilized a modified vaccinia virus that expresses a CD4^+^ T-cell epitope-rich C-terminal fragment of EBNA1 along with the full-length sequence of LMP2 [150, 151]. This vaccine successfully induced CD4^+^ and CD8^+^ EBV-specific T cells in a dose-dependent manner. For HCC, HBV and HCV are notorious for inducing liver malignancy and disrupting immune homeostasis, whereas HBC/HCV-specific vaccines might have the potential to reverse immune dysregulation [152, 153].

### Gut microbial metabolites

Gut microbial metabolites enhance cancer immunotherapy efficacy through modulating host antitumor immunity. Strategies leveraging FMT or engineered probiotics to enrich beneficial metabolites, such as SCFAs, tryptophan derivatives, and inosine, can reprogram the immunosuppressive TME and synergize with ICB. These promising results position microbial metabolites as therapeutic adjuvants to optimize cancer immunotherapy.

#### Short-chain fatty acids

SCFAs, primarily acetate, propionate and butyrate, are generated through microbial fermentation of dietary fibers. SCFAs critically maintain gut barrier integrity and intestinal homeostasis through activating GPRs (mainly GPR43, GPR41, and GPR109A) and inhibiting the epigenetic regulators HDACs [154]. Numerous studies have reported that supplementation with SCFA-producing probiotics suppresses tumor development in both preclinical mouse models and human patients and that SCFAs can activate cytotoxic T cells while inhibiting immunosuppressive cells. For example, microbe-derived butyrate directly potentiates the antitumor CD8^+^ T-cell response through an inhibitor of differentiation 2 (ID2)-dependent mechanism, increasing therapeutic efficacy in CRC mouse models [155]. A prospective study of 52 patients with solid tumors revealed that elevated fecal levels of acetate, propionate, butyrate, and valerate are associated with prolonged PFS and better treatment response in patients receiving anti-PD-1 therapy [111, 156]. Consistent findings were observed in patients with NSCLC receiving anti-PD-1 therapy [157].

Several studies have investigated the clinical benefits associated with the direct supplementation of SCFAs and/or SCFA-producing bacteria. Butyrate-producing *Clostridium butyricum CBM588* can modulate the gut microbiota by increasing the abundance of beneficial bacteria, including *Lactobacilli*, *Lactococcus*, and *Bifidobacteria*, and promoting the expansion of IL-17A^+^ γδT cells and IL-17A^+^ CD4 + T cells in the colon lamina propria [158]. Compared with β-lactam and quinolone-based antibiotic therapy, Tomita and colleagues reported that combining anti-PD-1 (nivolumab/pembrolizumab) or anti-PD-L1 (atezolizumab) with *C. butyricum CBM588* significantly improved the ICB response in patients [159]. In line with these findings, a phase 1 trial (NCT03829111) of metastatic RCC revealed that patients receiving a combination of nivolumab (anti-PD-1) plus ipilimumab (anti-CTLA-4) with *C. butyricum CBM588* have a greater ICB response rate and PFS [160]. Compared with those without *C. butyricum CBM588*, these patients also have greater abundances of beneficial gut commensals (e.g., *B. longum* and *Butyricimonas faecalis*) and circulating proinflammatory cytokines (e.g., IL-1β, G-CSF, GM-CS, and MCP-1).

However, there is controversy regarding the role of SCFAs in cancer immunotherapy. Clélia Coutzac and colleagues reported that high systemic butyrate and propionate levels are correlated with CTLA-4 blockade resistance and an elevated population of immunosuppressive Tregs, suggesting that SCFAs may constrain CTLA-4-induced antitumor activity [161]. Similarly, Wang and colleagues revealed that acetate supplementation accelerates tumor development while impairing CD8^+^ T-cell infiltration, whereas these effects are reversed after blocking acetate uptake [162]. Mechanistically, acetate-derived acetyl-CoA induces c-Myc acetylation and stabilizes oncoproteins, subsequently promoting the transcription of genes encoding PD-L1, glycolytic enzymes, monocarboxylate transporter 1, and cell cycle regulators.

#### Tryptophan metabolites

Tryptophan (Trp) is an essential amino acid obtained from dietary sources. It is utilized primarily through three metabolic pathways involving the enzyme indoleamine 2,3-dioxygenase for protein synthesis, serotonin production, and conversion into kynurenine. Tryptophan metabolites, including kynurenine, serotonin, and indole derivatives, play crucial roles in regulating the immune response and neurotransmission and maintaining intestinal homeostasis, and recent evidence has shown their potential as therapeutic targets in immunotherapy. Supplementation with the probiotics *L. johnsonii* and *L. gallinarum* is an effective strategy to increase ICB efficacy through tryptophan metabolism to modulate CD8^+^ T-cell stemness and Tregs, respectively [87, 102]. Daily administration of *L. reuteri* also contributes to increased ICB efficacy in mice, in which *L. reuteri* facilitates the translocation of gut microbes into melanoma tumors to trigger the function of antitumor IFN-γ^+^ CD8^+^ T cells [163]. Like other probiotic *Lactobacillus species*, intratumoral *L. reuteri* species mediate the metabolism of dietary tryptophan into indole-3-aldehyde (I3A) to activate AHR and subsequently increase the efficacy of ICB.

Huang and colleagues reported that ginseng polysaccharides enhance the efficacy of anti-PD-1 therapy by increasing microbe-derived valeric acid, decreasing kynurenine, suppressing Treg activity, and promoting the differentiation of effector T cells [164]. In head and neck squamous cell carcinoma (HNSCC), kynurenine secreted from tumor cells contributes to CD8^+^ T-cell exhaustion via accumulation mediated by intracellular transporters, whereas pharmacological blockade of these kynurenine transporters can restore cytotoxic T-cell function [165]. Mechanistically, kynurenine activates AHR to upregulate Siglec-15, which facilitates immune evasion by inhibiting T-cell infiltration and activation. Importantly, the protumorigenic effects of kynurenine on T-cell dysfunction are reversed by inhibiting Siglec-15, leading to increased anti-PD-1 therapy efficacy in mice with HNSCC. These results highlight that kynurenine is a therapeutic target for overcoming immunotherapeutic resistance [165].

#### L-arginine

L-arginine is a semiessential amino acid that plays a vital role in immune regulation and nitric oxide production, but its potential to predict immunotherapy response and improve treatment outcomes is increasingly recognized. L-arginine can reprogram global metabolism, leading to a metabolic shift from glycolysis to oxidative phosphorylation in activated T cells to promote the differentiation of central memory-like T cells [166]. A multicohort study investigated pretreatment plasma L-arginine levels in patients receiving anti-PD-1 therapy in 3 independent clinical trials (NCT02534649, NCT03984318, and NCT03000257) [167]. The results revealed that a low serum L-arginine concentration (less than 42 μM) was significantly associated with poor outcomes. Low serum L-arginine is also positively correlated with PD-L1 upregulation in myeloid cells, suggesting that L-arginine may serve as a predictive biomarker for the outcomes of ICB.

#### Inosine and purine metabolites

Inosine and purine metabolites are essential intermediates in energy metabolism and nucleotide biosynthesis and play pivotal roles in immune regulation and cancer development. In particular, inosine suppresses antitumor immunity by activating adenosine receptors on Tregs and MDSCs to facilitate tumor immune evasion and immune checkpoint blockade (ICB) resistance [112]. Purine metabolism promotes inflammation and tumor cell survival through oncogenic pathways such as the AMPK/mTOR pathway. The abnormal accumulation of purine metabolites such as hypoxanthine also induces oxidative stress under autoimmune conditions, while guanosine is implicated in supporting oncogenic signaling. Hence, targeting enzymes involved in purine biosynthesis, such as IMP dehydrogenase, has shown potential for increasing immunotherapy efficacy through reversing immunosuppression [168]. For example, a recent study revealed that the overexpression of adenosine deaminase, which converts adenosine to inosine, promotes stemness and enhances the functionality of CAR-T cells through metabolic reprogramming [169]. This reprogramming involves increased mitochondrial and glycolytic capacity, glutaminolysis, and polyamine synthesis, along with a reduction in glycolysis and epigenetic changes that favor CAR-T-cell stemness. Moreover, immunosuppression driven by purine metabolism in macrophages is linked to NLRP3/caspase-1/IL-1β signaling, whereas targeting this pathway could enhance antitumor immunity and the efficacy of anti-PD-L1 therapy in NSCLC [168]. In addition, Mager and colleagues showed that the probiotic *B. pseudolongum* improves immunotherapy efficacy through inosine production, but this process is dependent on ICB-induced dysfunction of the gut barrier, which allows systemic translocation of inosine and the subsequent activation of antitumor T cells [112].

#### Other emerging metabolites

Recent advances in metabolomic profiling technology have facilitated the discovery of novel metabolites that are associated with antitumor immunity and might increase ICB efficacy. For example, gut microbe-derived urolithin A enhances antitumor immunity by improving the persistence and effector functions of cytotoxic CD8^+^ cytotoxic T cells and human CAR-T cells [170, 171]. Mechanistically, urolithin A directly interacts with extracellular signal-regulated kinase (ERK)-1 and ERK2 to promote their activation, thereby activating antitumor T cells in mice with lymphoma and lung cancer. Microbial vitamin B5 (or pantothenic acid) is the precursor for coenzyme A, which promotes the differentiation of cytotoxic CD8^+^ T cells into IL-22-producing Tc22 cells, which exhibit robust antitumor activity and are linked to an improved immunotherapy response [172]. Vitamin B5 also directly enhances the efficacy of anti-PD-L1 therapy in mice, while a higher pretreatment level of plasma pantothenic acid is associated with a better response to anti-PD-L1 therapy in patients with MM [173]. Moreover, gut microbe-derived trimethylamine N-oxide (TMAO) can be utilized as an adjuvant in ICBs. Previous studies reported that *Enterococcus* and *Clostridium* are the major producers of trimethylamine, which is the precursor of TMAO and is associated with improved survival in cancer patients [174]. Mechanistically, TMAO activates the protein kinase R-like ER kinase (PERK) pathway, resulting in endoplasmic reticulum stress and initiating gasdermin-E (GSDME)-mediated pyroptosis, which in turn increases CD8^+^ T-cell-mediated antitumor immunity [175].

### Prebiotics and diets

Dietary strategies that aim to modulate the gut microbiota for health benefits, such as prebiotic supplementation and customized dietary plans, are gaining recognition for their potential to improve the efficacy of immunotherapy.

#### Prebiotics and polyphenols

Prebiotics, such as inulin and resistant starch, are nondigestible fibers that nourish beneficial gut bacteria, promoting digestion, immunity, and metabolic health. Found in foods such as garlic, onion, and bananas, they help maintain the gut microbiota balance and support overall well-being. A recent study using various murine tumor models demonstrated that an orally administered colon-retentive gel formulation of inulin effectively modulates the gut microbiota in situ, stimulates systemic memory T-cell responses, and enhances the antitumor efficacy of α-PD-1 checkpoint blockade. This enhancement occurs through the promotion of key commensal microorganisms and their short-chain fatty acid metabolites while facilitating the development of stem-like TCF-1^+^PD-1^+^CD8^+^ T cells within the tumor microenvironment [176]. Notably, in C57BL/6 mice, inulin administration produced a stronger antitumor effect than did anti-PD-1 alone or in combination. Analysis of tumor-infiltrating lymphocytes revealed increased IFN-γ production by γδ T cells and CD4^+^ cells in the presence of inulin compared with anti-PD-1 monotherapy [177]. Additionally, Change et al. reported that dietary prebiotic starches, which increase butyrate production, represent a promising strategy for microbial manipulation to alleviate ICI-induced colitis and improve patient outcomes [178].

Polyphenols, which are abundant in plant-based foods, act as antioxidants and anti-inflammatory agents, scavenging free radicals and influencing the composition of the gut microbiota. Recent evidence underscores the synergistic effects of polyphenols and prebiotics. Polyphenols may enhance the efficacy of prebiotics by selectively promoting beneficial bacterial species, while prebiotics can improve the bioavailability of polyphenols through microbial transformation. Together, these factors contribute to reduced inflammation, improved gut barrier function, and protection against chronic diseases such as obesity, diabetes, and colorectal cancer. The incorporation of both compounds into the diet through sources such as garlic, onion, berries, green tea, and dark chocolate offers a comprehensive approach to microbiota-targeted cancer therapy [179]. Notably, researchers identified castalagin, an ellagitannin found in polyphenol-rich camu-camu berries, as a key active compound. Oral supplementation with castalagin-enriched bacteria, such as *Ruminococcaceae* and *Alistipes*, is associated with favorable immunotherapy responses and improves the CD8⁺/FOXP3⁺CD4⁺ ratio in the TME. Following FMT from patients unresponsive to ICI therapy into mice, castalagin supplementation enhances the efficacy of anti-PD-1 treatment [180].

#### Dietary patterns

Dietary patterns significantly influence the composition of the gut microbiota, which in turn affects immune responses that are crucial for the effectiveness of tumor immunotherapy. For example, a ketogenic diet, a high-fat, low-carbohydrate regimen, may impact tumor immunity by lowering glucose availability and increasing the levels of ketone bodies. These metabolic changes can inhibit tumor growth, increase T-cell function, and reshape the immunosuppressive TME. Dai et al. reported that dietary-induced changes in cellular bioenergetics enhance the efficacy of anti-CTLA-4 immunotherapy by downregulating PD-L1 expression and upregulating type I interferon and antigen-presentation pathways, leading to prolonged survival in syngeneic murine tumor models [181]. Other studies have shown that ketogenesis can improve ICB effectiveness through mechanisms such as intrinsic upregulation of MHC class I in cancer cells, increased recruitment of CD8^+^ T cells, polarization of M1 macrophages, differentiation of monocytes into APCs, and reduced neutrophil infiltration. These findings suggest that optimizing ketogenic diets may enhance ICB outcomes in prostate cancer patients [182].

#### Nutritional supplements

Omega-3 fatty acids, which are found in sources such as fish, nuts, and seeds, are beneficial for the heart, brain, and inflammatory diseases because they reduce inflammation, increase cholesterol levels, and support cellular function. King et al. revealed that an increased PUFA/SFA ratio is associated with increased sensitivity to anti-PD-1 treatment in mouse models of colorectal, urothelial and lung cancer [183]. To support this finding, supplementation with docosahexaenoic acid (DHA), an ω-3 polyunsaturated fatty acid, has been shown to increase the effectiveness of PD-1 blockade in humanized NSCLC immune-xenograft models. Mechanistically, increasing PUFA levels in cell membrane phospholipids impaired PD-1/PD-L1 binding by altering the plasma membrane composition and fluidity, thereby improving the responsiveness to ICB [184].

Vitamin D levels are regulated by the blood carrier protein group-specific component (Gc) globulin. In intestinal epithelial cells (IECs), 1,25-dihydroxyvitamin D3 [1,25-(OH)₂D₃] binds to the nuclear vitamin D receptor (VDR), a widely expressed ligand-activated transcription factor, to regulate the expression of vitamin D-responsive genes [185]. Emerging research underscores the essential role of vitamin D in regulating the immune system under both normal and pathological conditions [186]. In a study by Giampazolias et al., a Gc-deficient mouse model with low blood vitamin D was used to investigate whether Gc suppresses anticancer CD8⁺ T-cell responses in transplantable tumors. These findings revealed that Gc deficiency enhanced CD8⁺ T-cell-mediated tumor control and improved the efficacy of immunotherapy. Additionally, the binding of 1,25-(OH)₂D₃ to the VDR in IECs promotes a microbiota rich in *Bacteroides fragilis*, which can inhibit tumor development and enhance anticancer immunotherapy in mice [187].

### Genetically engineered bacteria

Synthetic biology tools enable the precise engineering of bacterial strains to specifically target neoplastic tissues, modulate host immune responses, and deliver therapeutic cargos. Current research focuses on spatially confined therapeutic strategies and combinatorial treatment approaches. For example, clinical trial results highlight the potential of engineered *Listeria monocytogenes* strains in cancer immunotherapy. In a phase II study involving 90 patients with previously treated metastatic PDAC, a dual vaccination strategy combining CRS-207 (an engineered *Listeria monocytogenes* strain that secretes mesothelin), GVAX (a GM-CSF-secreting allogeneic PDAC cell line vaccine), and low-dose cyclophosphamide resulted in longer overall survival than did GVAX plus cyclophosphamide alone, with minimal toxicity [188]. Additionally, another phase II trial with 93 patients who had previously been treated for metastatic PDAC revealed that incorporating nivolumab into the CRS-207/cyclophosphamide/GVAX regimen restructured the tumor microenvironment by increasing CD8^+^ T-cell proliferation and decreasing TAM and myeloid cell populations. This reprogramming of the tumor microenvironment was associated with prolonged survival and was not observed in patients who did not receive nivolumab [189]. Furthermore, data from ongoing phase I/II trials (NCT03847519) indicate that ADXS-503, an engineered *Listeria monocytogenes* strain expressing 22 antigens associated with NSCLC, achieved durable disease control in 46–67% of patients with either squamous or nonsquamous NSCLC when combined with pembrolizumab [190]. Recently, Chen and colleagues developed an engineered skin bacterial strain of *Staphylococcus epidermidis*, designated NIHLM087, which expresses melanoma tumor antigens and promotes the infiltration of antitumor T cells. This engineered *S. epidermidis* is safe and easy to use, yielding a potent antitumor response when combined with ICB therapy in both localized and metastatic melanoma [191]. Overall, engineered bacteria enhance tumor responsiveness to ICB by remodeling the TME through improved antigen presentation and targeted delivery of immune modulators, reducing T-cell exhaustion, and potentially overcoming resistance by engaging multiple immune pathways.

### Oncolytic virus immunotherapy

Oncolytic virus immunotherapy utilizes engineered viruses to specifically target and destroy cancer cells through two main mechanisms: direct lysis and immune activation. Unlike traditional therapies, these agents selectively exploit tumor-specific pathways instead of merely targeting replicating cells, which diminishes reliance on receptor expression and helps overcome mutational resistance. This approach enhances preexisting antitumor immunity or generates new antigen responses, ultimately reshaping the tumor microenvironment.

Early trials, especially those combining oncolytic viruses with checkpoint inhibitors, have shown promising results by promoting systemic antitumor effects and transforming treatment paradigms through more precise and safer interventions. Recent studies have indicated that combining oncolytic viruses with other therapies increases the expression of proinflammatory cytokines, including IFN-γ, which activates JAK1/2 signaling and improves antigen presentation, thereby increasing tumor sensitivity to checkpoint blockade [192, 193]. Additionally, early-phase trials have shown that pairing oncolytic viruses with CTLA-4 or PD-1 inhibitors yields clinically beneficial outcomes, often surpassing the efficacy of single-agent therapies. For example, Puzanov et al. reported the safety and therapeutic potential of talimogene laherparepvec (T-VEC), an oncolytic virus, when combined with ipilimumab (a CTLA-4 inhibitor) in patients with advanced melanoma. This open-label, multicenter phase Ib study (NCT01740297) demonstrated manageable adverse effects and better outcomes for the combination than for the monotherapies [194].

However, the effectiveness of oncolytic viruses in cancer immunotherapy has demonstrated trial-to-trial variability. The abovementioned phase Ib study assessing T-VEC combined with pembrolizumab (a PD-1 inhibitor) in patients with unresectable stage IIIB-IV melanoma revealed significant clinical benefits, as evidenced by improved objective response rates (ORRs) and complete response (CR) rates. In contrast, data from a subsequent phase III trial revealed that the combination of T-VEC and pembrolizumab did not increase PFS or OS compared with placebo plus pembrolizumab. These contrasting results highlight the necessity for further mechanistic optimization and refined patient selection to maximize the therapeutic synergy between oncolytic virotherapy and ICB therapy.

### Bacteriophage therapy that targets gut pathobionts

Gut bacteriophages (termed phages hereafter) are a group of viruses that specifically target bacteria or microbiota-encoded genes without altering the overall microbiota composition [195]. Like prokaryotic viruses, phages primarily consist of nucleic acid and capsid structural components [196, 197]. Numerous studies have confirmed the biosafety profile of phages because of their exclusive specificity for bacterial infection, with no interaction with eukaryotic cells [198–200]. The target range of phages varies greatly. For example, the JHP phage has polyvalent infectivity against a broad spectrum of bacteria, including *Pseudomonas aeruginosa*, *E. coli*, *Salmonella enterica*, *Campylobacter jejuni, Acinetobacter baumannii*, and *Proteus mirabilis*, whereas the M13 phage specifically targets *E. coli* [201].

M13 phage are classified as filamentous phages that can directly activate the TLR signaling pathway to induce an adaptive immune response through their deoxycytidylate-phosphate-deoxyguanylate (CpG) motifs in genomic DNA [202, 203]. The intrinsic PAMPs of M13 phages also allow rapid recognition by APCs to effectively prime the innate immune response [204, 205]. Owing to their potent immunogenicity, biosafety advantages, environmental stability, and convenience in storage, M13 phages have emerged as excellent candidates for developing precision vaccine platforms. A standardized M13 phage-based vaccine platform has been designed to display specific antigenic epitopes for cancer immunotherapy [206, 207].

Another example of a phage with therapeutic potential is the engineered *E. coli*-targeting phage SNIPR001 [208]. Gencay and colleagues designed this phage to specifically target intestinal *E. coli* that tends to undergo bloodstream translocation in patients with neutropenic hematologic cancer, where such bacterial migration can trigger life-threatening bloodstream infection. An ongoing phase 1 clinical trial (NCT05277350) in the US is systematically assessing the safety profile of SNIPR001 and its ability to reduce *E. coli* while sparing the gut microbiota. The results demonstrated its potential to mitigate bacterial translocation and the consequent risk of bacteremia during oncologic therapy. Hence, this phage shows great promise in reducing the toxicity of cancer therapy and improving overall treatment efficacy.

## Multikingdom microbial biomarkers in predicting immunotherapy response

The human gastrointestinal tract harbors over 1000 microbial species from various kingdoms, including bacteria, prokaryotes, viruses, protists, and fungi [114]. While current immunotherapy research has focused on bacterial components of the gut microbiota, the roles and functions of nonbacterial gut microbes, particularly the fungal mycobiota, in ICB are largely unexplored. Although the fungal mycobiota constitutes less than 0.1% of the gut microbiota, it markedly impacts the microbial balance and bacterial communities in the human gut [209–211]. Emerging evidence also underscores the involvement of the fungal mycobiota in cancer development, immunomodulation, bidirectional interactions with gut bacteria and host metabolism, and ICB efficacy across different cancer types [212, 213]. A prospective study characterized the gut microbiota, mycobiota, and metabolome in 80 patients with advanced HCC receiving ICB therapy [214]. The bacterial and metabolomic profiles of patients with durable clinical benefits are distinct from those of patients without benefits; however, such differences are not significant for the fungal mycobiota. Higher microbial diversity before treatment is associated with better clinical benefits, with notable differences observed at 6–8 weeks after treatment. On the other hand, specific fungal species, such as *Actinomyces* sp. *ICM47* and *Senegalimassilia anaerobia*, are linked to improved patient survival. These multikingdom biomarkers therefore emphasize the crucial role of nonbacterial gut microbes in determining the efficacy of cancer immunotherapy.

In the gut microbiota, bacteria and fungi coexist in a complex and densely populated microenvironment where neither kingdom functions in isolation. Recent findings have revealed that dynamic cross-kingdom interactions influence essential biological processes, such as biofilm formation, metabolic pathways, population dynamics, and the integrity of the gut barrier. These interdependent relationships suggest that fungal‒bacterial interactions are critical in shaping gut homeostasis and the host immune response. In light of this, a study analyzed 862 fecal metagenomes from 9 independent cohorts to assess the contribution of fungi to the ICB response. While fungal biomarkers alone have a strong ability to predict the ICB response, their performance is further improved after the incorporation of bacterial biomarkers [215]. Fungal biomarkers are also associated with responders who have relatively high levels of exhausted T cells, while functional analysis revealed enrichment of SCFA-producing *Schizosaccharomyces octosporus* in ICB responders. In another multicohort study, the impacts of the gut microbiota on ICB efficacy across multiple cancer types (MM, NSCLC, HCC, and renal cell carcinoma) were examined. Using a multikingdom approach to characterize microbial signatures predictive of therapeutic outcomes, this study identified responder-enriched bacteria (e.g., *Faecalibacterium prausnitzii*, *Coprococcus comes*), eukaryotes (e.g., *Nemania serpens*, *Hyphopichia pseudoburtonii*), and a depletion of *Hungatella hathewayi* in at least two cancer types [216]. Functional studies further demonstrated that these microbes could modulate CD8^+^ T-cell activity in vitro and in mice. Moreover, a panel of combined bacterial and fungal biomarkers achieves better predictive performance than panels of only bacterial or fungal biomarkers do, hence confirming the importance of the transkingdom microbiota in the ICB response.

In addition to microbes, their derived metabolites also play crucial roles in mediating the ICB response. A recent study by Zhu and colleagues performed integrative multiomics analysis of the fecal microbiota and metabolome in ICB-treated patients from multiple independent cohorts [217]. The study identified 20 bacteria that are positively associated with therapeutic outcomes, such as *Adlercreutzia equolifaciens*, *Allisonella histaminiformans*, *Eubacterium ramulus*, *Holdemania filiformis*, *Blautia obeum/wexlerae*, *F. prausnitzii*, and *Bacteroides massiliensis*. In contrast, *Klebsiella pneumoniae* and *Klebsiella michiganensis/oxytoca* were negatively correlated with the clinical response. Simultaneously, metabolomic profiling revealed several fecal metabolites that are linked to the ICB response, including D-ribose, methionine sulfoximine, uracil, vitamin B2,7-DHCA, and phenylacetylglutamine (PAGln). Among these metabolites, the responder-enriched *Eubacterium ramulus* had a significant positive correlation with the fecal vitamin B2 level, whereas *K. pneumoniae*-derived PAGln was associated with ICB resistance. Preclinical validation demonstrated that PAGln administration accelerated tumor progression and reduced ICB efficacy in mice with CRC. Mechanistically, the combination of PAGln and ICB results in a decreased proportion of effective CD8^+^ T cells and increased number of exhausted CD8^+^ T cells, suggesting that PAGln may impair the antitumor T-cell response to diminish ICB efficacy. Hence, this study provides insights into how the crosstalk between gut microbes and their metabolites can be leveraged to predict immunotherapy response and patient prognosis.

## The gut microbiota in alleviating the side effects of cancer immunotherapy

### Microbial signatures in immune-related adverse events

The application of ICB is limited by immune-related adverse events (irAEs), ranging from mild skin reactions to life-threatening pneumonitis, which affects nearly 70% of patients. Recent research has illustrated the pivotal role of gut microbes in mediating immunotherapy-related toxicity. For example, a study reported that 49% of ICB-treated patients with advanced MM experience ≥ grade 3 irAEs, whereas *Bacteroides intestinalis* is enriched in these patients and correlated with mucosal IL-1β upregulation [218]. McCulloch and colleagues identified two distinct microbial clusters (Lachnospiraceae and *Streptococcus*) with opposite effects on ICB outcomes and the occurrence of irAEs, of which *Lachnospiraceae* species are independently associated with immune-related toxicity [219]. In a phase III trial (JCOG2007), microbiota analysis revealed that lower alpha diversity is linked to an increased risk of severe (≥ grade 4) irAEs, whereas beneficial *Fusicatenibacter* and *Butyricicoccus* are associated with a reduced likelihood of severe adverse events [220]. Similarly, Liu and colleagues reported that decreased microbial diversity is correlated with a greater risk of irAEs in ICB-treated patients [221]. In particular, severe irAEs (grade ≥ 3) are associated with enriched *Streptococcus*, *Paecalibacterium*, and *Stenotrophomonas*, whereas ICB-treated patients with mild or no irAEs have a greater abundance of *Faecalibacterium*. In addition, different irAEs were found to be correlated with distinct microbial taxa [221]. For example, patients with ICB-induced thyroid dysfunction have increased *Paecalibacterium* abundance and decreased abundance of *Bacteroides* and *Lactobacillus*, whereas those with severe diarrhea are characterized by the enrichment of *Stenotrophomonas* and *Streptococcus*.

### Treatment strategies

#### Probiotic supplementation

Targeting the gut microbiota can alleviate ICB-induced irAEs. A study by Gao and colleagues revealed that *F. prausnitzii* supplementation enhances antitumor immunity in ICB-treated mice with CRC, accompanied by reduced severity of ICB-induced colitis [222]. Mechanistic analysis revealed that the beneficial effects of *F. prausnitzii* are associated with alterations in the gut microbiota and increased microbial alpha diversity, leading to marked expansion of beneficial commensals and depletion of pathogenic bacteria. Similarly, Wang and colleagues reported that *Bifidobacterium* effectively alleviates colitis-induced CRC in mice by modulating the metabolic function of Tregs without noticeably compromising antitumor immunity [223].

#### Fecal microbiota transplantation

Emerging evidence has positioned FMT as a promising therapeutic strategy for managing ICB-induced colitis. Shang and colleagues reported that transplanting stool from immunotherapy-related colitis patients prior to disease onset induces a stronger colitogenic response in recipient mice, suggesting that microbial signatures are potential biomarkers for predicting and mitigating the risk of irAEs during immunotherapy [224]. Wang and colleagues reported successful FMT to treat refractory ICB-associated colitis, leading to the restoration of the gut microbiota and mucosal immunity [13]. FMT also markedly reduces inflammation and resolves ulcers, accompanied by a significant decrease in proinflammatory CD8^+^ T cells and an increase in CD4^+^FoxP3^+^ Tregs in the colonic mucosa. These findings thus imply that FMT alleviates ICB toxicity by rebalancing the gut microbiota-associated immune response.

#### Prebiotics and dietary modulation

Fibers and omega-3 fatty acids are dietary components that can mitigate irAEs. Simpson and colleagues reported a significant correlation between dietary nutrients and the gut microbiota characterized by microbial diversity, homeostatic function, and therapeutic response without severe toxicity. In particular, Ruminococcaceae and Bacteroidaceae are associated with high intake of these nutrients, and these taxa can enhance the antitumor immune response while simultaneously reducing the incidence of irAEs in MM patients receiving neoadjuvant ICB [15].

## Future perspectives

The integration of microbiota profiling into precision oncology frameworks could reshape immunotherapy paradigms. Personalized therapeutic approaches, such as antibiotics or FMT protocols targeting *H. pylori* and *B. fragilis* for CRC, have shown significant promise in improving treatment efficacy [225–227]. Simultaneously, multiomics monitoring platforms utilizing stool and blood samples would facilitate real-time adaptive treatment modifications. The use of cryopreserved microbial consortia, combined with protocols for selecting HLA-matched donors, would ensure standardized therapeutic delivery. Moreover, precision FMT methodologies that utilize strain-specific microbial consortia would help reduce interpatient variability, thereby increasing the therapeutic precision of microbiota-based interventions [228, 229].

Future perspectives targeting the gut microbiota in cancer immunotherapy should focus on artificial intelligence (AI) technology. Through integrative metagenomic, transcriptomic, and metabolomic analyses, novel host‒microbe interactions and molecular pathways can be identified. AI algorithms also have the potential to identify specific microbial signatures, such as the overabundance of *Streptococcus*, which is associated with ICB resistance in NSCLC and may be utilized in the development of novel and improved therapies [230, 231]. Additionally, advanced single-cell resolution mapping of host‒microbe interactions would allow deeper elucidation of the mechanistic process by which beneficial gut commensals and/or probiotics (e.g., *Bifidobacterium*) enhance CD8^+^ T-cell infiltration into tumors [232]. These innovative platforms might enable precise targeting of immunosuppressive niches, with spatially resolved intervention strategies providing potential solutions to treatment resistance.

Emerging evidence has revealed distinct microbial patterns across populations. For example, rural communities that consume high-fiber diets have a greater abundance of SCFA-producing taxa, which may explain the greater ICB response in non-Western populations [233]. Conversely, industrialized diets rich in processed fats are associated with dysbiosis dominated by *Bacteroides*, potentially attenuating immunotherapy efficacy [234]. In addition to geographic factors, biological variables also significantly influence therapeutic outcomes. Aging-related decreases in microbial diversity and butyrate production can impair ICB responsiveness in elderly patients [235, 236]. Moreover, several microbial species are associated with the regulation of sex hormones (e.g., *Prevotella*), thus highlighting the potential need for developing and optimizing sex-specific probiotic strategies to improve treatment efficacy [237, 238].

## Conclusion

In summary, the gut microbiota serves as a master regulator of cancer immunity, dynamically orchestrating immunotherapy responsiveness through bidirectional host‒microbe interactions. Pathogenic bacteria such as *H. pylori* and ETBF drive tumorigenesis through inducing chronic inflammation, recruiting immunosuppressive cells (e.g., Tregs and MDSCs), and upregulating immune checkpoints. Conversely, beneficial gut commensals, including *A. muciniphila* and *Bifidobacterium*, potentiate ICB efficacy by activating DCs, increasing CD8^+^ T-cell infiltration, and polarizing macrophages toward antitumor phenotypes. The gut microbiota also influences ICB efficacy by shaping T-cell cytotoxic functionality and cytokine profiles. Microbe-derived metabolites such as SCFAs and tryptophan derivatives, as well as PRR ligands (e.g., LPS), prime DCs to increase the intratumoral infiltration of CD8^+^ T cells. Clinically, microbiota-targeting strategies (e.g., FMT, probiotics, engineered bacteria) have demonstrated translational potential in enhancing ICB responsiveness and mitigating irAEs. Moreover, multikingdom microbial signatures in addition to bacteria (e.g., fungi and viruses) are being explored as prognostic biomarkers for treatment response. Nevertheless, despite these advancements, challenges persist, including interpatient variability in microbiota composition, inconsistent intervention protocols, and an incomplete understanding of host‒microbe interactions in the TME. The combination of personalized microbial therapeutics, AI-driven multiomics stratification, and adaptive trial designs in future studies might help to decode microbiota‒drug synergies, thereby accelerating bench-to-bedside translation.
